# Unfavorable Associations Between Serum Trimethylamine N-Oxide and L-Carnitine Levels With Components of Metabolic Syndrome in the Newfoundland Population

**DOI:** 10.3389/fendo.2019.00168

**Published:** 2019-03-26

**Authors:** Xiang Gao, Yuan Tian, Edward Randell, Haicheng Zhou, Guang Sun

**Affiliations:** ^1^College of Life Sciences, Qingdao University, Qingdao, China; ^2^Faculty of Medicine, Memorial University, St. John's, NL, Canada; ^3^Xiangyang Central Hospital, Affiliated Hospital of Hubei University of Arts and Science, Xiangyang, China; ^4^The Department of Endocrinology, the First Affiliated Hospital of Dalian Medical University, Dalian, China

**Keywords:** L-carnitine, TMAO, metabolic syndrome, insulin resistance, cross-sectional

## Abstract

**Background:** We aimed to study the relationships between serum Trimethylamine N-oxide (TMAO) and L-carnitine levels with metabolic syndrome profiles, including obesity, blood pressure, serum lipids, serum glucose and insulin resistance (IR)-related index in humans.

**Methods:** Cross-sectional study was performed in 1,081 subjects from the CODING study in Newfoundland. Serum TMAO and L-carnitine levels were quantified by LC-MS/MS. Metabolic markers were measured in all subjects using fasting blood samples. Partial correlation and linear regression analysis were employed after systematically controlling the major confounding factors, such as age, gender, calorie intake and physical activity level.

**Results:** Serum L-carnitine level was positively correlated with serum triglyceride (TG), serum insulin, IR in males with normal fasting glucose (*p* < 0.05 for all) and positively correlated with only serum TG (*p* < 0.05) in those with hyperglycemia. In females, significant positive correlations were identified between serum L-carnitine level with obesity, serum total cholesterol, glucose, insulin, and IR in those with normal fasting glucose level (*p* < 0.05 for all), while none was found in those with hyperglycemia. Serum TMAO level was only identified to be positively correlated with serum insulin level and IR in hyperglycemic males (*p* < 0.05 for all).

**Conclusions:** Serum L-carnitine level was significantly associated with an unfavorable metabolic syndrome (MS) profile mainly in subjects with normal serum glucose level, while serum TMAO level was associated with an unfavorable MS profile in subjects with hyperglycemia. The gender difference warrants further investigations.

## Introduction

Trimethylamine N-oxide (TMAO) is a product of trimethylamine (TMA) oxidation. Recently, TMAO has been recognized as a novel risk factor for cardiovascular diseases and reported to be elevated in several cardiovascular events and type 2 diabetes mellitus (T2DM) (1, 2). In humans, TMAO is acquired from either direct uptake from TMAO containing foods, especially seafood, or *in vivo* production from its metabolic precursors, like L-carnitine, choline, and betaine (1). L-carnitine is an essential nutrient involved in lipid metabolism, which can be biosynthesized from amino acids, specifically L-lysine and L-methionine, or directly obtained from foods, such as beef, lamb, fish, poultry and milk (3). As an important precursor for TMAO, dietary L-carnitine can be metabolized by gut bacteria to release TMA, which is subsequently oxidized in the liver by flavin-containing monooxygenase 3 to form TMAO (4).

Metabolic syndrome (MS) is defined by a constellation of multiple risk factors for T2DM and cardiovascular disease (CVD), including hyperglycemia, dyslipidemia, obesity, hypertension and impaired insulin sensitivity (5). Currently, MS affects ~30% of individuals across the world and has become one of the major worldwide public-health challenges (6). The mechanism of MS is not clear yet but insulin resistance (IR) and obesity may play critical roles (7). Evidence for an effect of TMAO and L-carnitine on MS can be found in both animals and humans. TMAO was first reported to be harmful to human health in 2011 (8). Wang et al. found that serum TMAO level was positively related with the risk of CVD in humans and proved that dietary TMAO supplementation promoted atherosclerosis in mice (8). Subsequent studies confirmed that serum TMAO levels positively correlated with CVD events (9). Other studies also proved that serum TMAO levels were elevated in T2DM patients (10). In a previous study, we found that dietary TMAO exacerbated IR in high fat diet-fed obese mice (11). A recent cross-sectional study in 300 patients with increased cardiovascular risk indicated that serum TMAO level was positively correlated with IR (12). L-carnitine, which can be metabolized to TMA, is a primary metabolic precursor for TMAO. Dietary L-carnitine supplementation can significantly increase serum TMAO levels in both human and rodents (4, 13). Oral L-carnitine treatment could also promote atherosclerosis in ApoE knockout mice (10). L-carnitine is well-known as a critical long fatty acid transport facilitator involved in lipid metabolism, especially fatty acid oxidation. Numerous studies in both humans and animals also show that dietary L-carnitine could notably reduce body weight and improve serum lipid profiles (14, 15). Serum L-carnitine levels are significantly lower in obese pregnant women (16). L-carnitine can also alleviate IR in high fructose-fed rodents (17), while evidence of the same in humans is lacking. Collectively, these reports indicate a potential relationship between serum TMAO and L-carnitine levels with MS and its risk factors. Moreover, due to the production of TMAO, the beneficial effect of L-carnitine on health is controversial.

To our knowledge, no previous study has systematically examined the relationships of serum TMAO or L-carnitine level with MS components in humans. To fill this knowledge gap, the present cross-sectional study was performed to clarify the association of serum TMAO and L-carnitine levels with MS related risk factors, including obesity, serum lipids, serum glucose, blood pressure, and IR-related index in Newfoundland population. Our findings provide new insights into the relationships between TMAO and its metabolic precursors in human health.

## Materials and Methods

### Study Population

One thousand and eighty-one subjects from the CODING study in the Newfoundland province of Canada were enrolled (18, 19).

This study was carried out in accordance with the recommendations of the Health Research Ethics Authority (HREA), Memorial University, St. John's, NL, Canada, with Project Identification Code #10.33 (latest date of approval: 11 February 2016) with written informed consent from all subjects. All subjects gave written informed consent in accordance with the Declaration of Helsinki. The protocol was approved by the HREA (20, 21).

### Anthropometric Measurements

After a 12-h fast, anthropometrics measurements were examined. Body weight, height, waist circumference (WC) and hip circumference of the participants were measured as described previously (20). Waist-to-hip ratio (WHR) was calculated by division of WC by hip circumference. BMI (kg/m^2^) was also calculated. Blood pressure of the subjects was measured by Dinamap 845 XT equipment after 10 min of resting.

### Lifestyle and Dietary Intake Assessment

Subjects' lifestyle-related information were collected with self-administered screening questionnaires, involving age, gender, and family origin, disease status, medicine use, alcohol consumption, smoking status, and menopausal status (for women only). Physical activity level was assessed by an ARIC Baecke Questionnaire (21).

Dietary intake was measured by a semi-quantitative Willett Food Frequency Questionnaire (FFQ) and a NutriBase Clinical Nutrition Manager software was used to obtain the total daily calorie intake (22).

### Biochemical Measurements

Venous blood samples were collected and serum samples were separated. Serum concentrations of glucose, triglycerides (TG), total cholesterol (TC) and high-density lipoprotein cholesterol (HDL-c) were detected on an Architech C16000 clinical chemistry analyzer (Abbott Laboratories, IL, USA). Low-density lipoprotein cholesterol (LDL-c) and atherogenic index (AI) were calculated by: LDL-c = TC - HDL-c - TG/2.2 and AI = (TC − HDL-c)/HDL-c (23), respectively. An Architect I2000 immunoassay analyzer was used to determine fasting insulin (FINS) level. IR and β cell function were assessed with homeostasis model assessment (HOMA) (24):

HOMA-IR = (Fasting Insulin [mU/L] × Fasting Glucose [mmol/L])/22.5
HOMA- β = (20 × Fasting Insulin [mU/L])/(Fasting Glucose [mmol/L] -3.5)
Quantitative insulin-sensitivity check index (QUICKI) was also calculated (25):
QUICKI = 1/[log (fasting insulin (mU/L)) + log (fasting plasma glucose (mmol/L) × 18.0182)].

### Serum TMAO and L-Carnitine Levels Measurements

Levels of serum TMAO and L-carnitine were detected by liquid chromatography with tandem mass spectrometry (LC/MS/MS). Detailed methods were described in our previous publication (26). Briefly, protein-free serum enriched with d_9_-TMAO and d_11_-L-carnitine (Cambridge Isotopes, Tewksbury, MA, USA) was injected into a normal-phase Atlantis HILIC Silica HPLC column (3 μM, 2.1 × 100 mm, Waters Corporation, Milford, MA). TMAO and L-carnitine were eluted in an isocratic solvent system consisting of 25% ammonium formate (15 mM, pH 3.0) and 75% acetonitrile and delivered to the tandem Micromass Ultima Triple-Quad mass spectrometer (Waters Corporation, Milford, MA). The m/z transitions during mass spectrometry were: d_9_-TMAO 85>68, TMAO 76>58, d_11_-L-carnitine 171.1>103, L-carnitine 162>103.

### Data Analysis

All data are presented as means ± SD (standard deviation). To normalize the data distributions, serum TMAO, fasting insulin, fasting glucose, serum TG, HOMA-IR, HOMA-β, and total calorie intake were log-transformed. Differences in measurements between women and men were detected by independent Student's *t-*test.

Pearson correlation analysis was performed to find potential confounding factors by studying the associations with MS profiles and serum TMAO or L-carnitine levels. Analysis of covariance (ANCOVA) were used to study the statistical interaction between serum TMAO, L-carnitine levels, and sex on the main outcomes. Partial correlation analysis was performed to evaluate the relationships of serum TMAO and L-carnitine levels with MS related indexes, including obesity, blood pressure, serum lipids levels, and IR related indexes within male and female groups. Age, total calorie intake, physical activity level, medicine status, alcohol intake, smoking status, and menopausal status (for females) were controlled during the analysis. Stepwise multiple linear regression analysis was performed to test the contribution of serum TMAO, L-carnitine levels to MS related components among women or men. MS-related components were used as dependent variables. Serum TMAO, L-carnitine levels and all the confounding factors were used as independent variables.

To further explore the associations between serum TMAO or L-carnitine levels with MS related components in different metabolic sub-groups, subjects were divided into normal or high serum glucose level groups based on the criteria of National Cholesterol Education Program (NCEP)-ATP III with fasting glucose ≥ 5.6 mmol/L recognized as hyperglycemia (27). Partial correlation analysis and stepwise multiple linear regression analysis were used to determine the correlations between serum TMAO and L-carnitine levels with MS profiles.

A SPSS 20.0 (SPSS Inc., Chicago, IL) software was used to assess all the statistical analyses. *P* < 0.05 was considered as statistically significant and all the tests were two sided.

## Results

### Demographic Parameters and Serum L-Carnitine, TMAO Levels

As shown in Table 1, male subjects were 2.7 years younger than female subjects (*p* < 0.001). Obesity measures, systolic pressure, diastolic pressure, physical activity level and dietary calorie intake were higher in males (*p* < 0.001 for all). For serum parameters, males had higher fasting TG and glucose levels, but lower serum HDL-c levels (*p* < 0.001 for all). Atherogenic index (AI), HOMA-IR index and HOMA-β index were dramatically lower, while QUICKI index was higher in females (*p* < 0.01 for all). Male subjects had significantly higher serum L-carnitine level (*p* < 0.001). No notable differences were found in hip circumference, serum TC, LDL-c, insulin, and TMAO levels between genders.

**Table 1:** Characteristics of the participants by gender.

	**Male (536)**	**Female (545)**	***P***
	**Mean ± SD**	**Mean ± SD**	
Age	42.21 ± 13.26	44.90+11.3	0.000
Weight (kg)	88.20 ± 15.67	70.3+13.2	0.000
BMI	28.27 ± 4.58	26.7+5.0	0.000
Waist circumference (cm)	99.45 ± 12.64	91.25+13.59	0.000
Hip (cm)	101.28 ± 9.94	102.53+11.42	0.055
Waist/Hip Ratio	0.98 ± 0.06	0.89 ± 0.07	0.000
Systolic pressure (mmHg)	132.66 ± 14.84	122.51 ± 16.14	0.000
Diastolic pressure (mmHg)	84.12 ± 10.17	80.42 ± 10.97	0.000
Physical activity level	8.37 ± 1.56	7.99 ± 1.49	0.000
Dietary calorie intake (kcal/day)	2201 ± 1021	1823 ± 787	0.000
Serum TG (mmol/L)	1.48 ± 0.99	1.17 ± 0.68	0.000
Serum TC (mmol/L)	5.08 ± 1.08	5.16 ± 0.97	0.199
Serum HDL-c (mmol/L)	1.19 ± 0.28	1.52 ± 0.38	0.000
Serum LDL-c (mmol/L)	3.22 ± 0.91	3.11 ± 0.90	0.057
Atherogenic index (AI)	4.42 ± 1.22	3.55 ± 0.97	0.000
Serum glucose (mmol/L)	5.32 ± 0.67	5.1 ± 0.67	0.000
Serum insulin (mU/L)	10.46 ± 7.06	9.66 ± 5.92	0.142
HOMA-IR	2.51 ± 1.81	2.24 ± 1.61	0.006
HOMA–β	399.11 ± 350.98	331.4 ± 339.1	0.000
QUICKI	0.35 ± 0.03	0.36 ± 0.032	0.009
Serum L-carnitine (μmol/L)	42.71 ± 9.85	36.77 ± 9.34	0.000
Serum TMAO (μmol/L)	6.15 ± 9.34	5.43 ± 7.09	0.177

*All values are mean ± SDs. BMI, body mass index; WC, waist circumference. Significant differences between female and male group, based on independence sample Student's t-test, Statistical significance was set to p < 0.05*.

### Correlation Between Serum L-Carnitine, TMAO Levels, and MS Related Indexes by Gender

The associations between serum L-carnitine and TMAO levels with MS related indexes in males and females after adjusting for confounding factors are presented in Table 2. In males, serum L-carnitine level was positively correlated with serum TG level (*r* = 0.139, *p* < 0.01), serum insulin level (*r* = 0.111, *p* < 0.05), HOMA-IR (*r* = 0.101, *p* < 0.05) and negatively correlated with QUICKI index (*r* = −0.104, *p* < 0.05). In females, serum L-carnitine level was positively correlated with weight (*r* = 0.186, *P* < 0.001), BMI (*r* = 0.178, *P* < 0.000), waist (*r* = 0.134, *P* < 0.01), hip (*r* = 0.171, *P* < 0.001), serum TC (*r* = 0.091, *p* < 0.05), glucose (*r* = 0.094, *p* < 0.05), insulin levels (*r* = 0.137, *p* < 0.01), HOMA-IR index (*r* = 0.143, *p* < 0.01), HOMA-β index (*r* = 0.134, *p* < 0.01), and negatively correlated with QUICKI index (*r* = −0.147, *p* < 0.001). No significant relationships were found between serum TMAO level and MS related risk factors.

**Table 2:** Partial correlations between serum L-carnitine, TMAO levels with components of MS in females and males.

	**Male**		**Female**	
	**r1(P)**	**r2(P)**	**r1(P)**	**r3(P)**
**L-CARNITINE (μmol/L)**				
Weight	0.019(0.652)	0.001(0.987)	0.197(0.000)	0.186(0.000)
BMI	0.062(0.152)	0.024(0.588)	0.225(0.000)	0.178(0.000)
Waist circumference	0.065(0.135)	0.007(0.866)	0.224(0.000)	0.134(0.002)
Hip circumference	0.081(0.060)	0.032(0.466)	0.239(0.000)	0.171(0.000)
Waist/hip ratio	0.009(0.828)	−0.031(0.473)	0.086(0.044)	0.005(0.903)
Systolic BP	0.071 (0.100)	0.041(0.351)	0.111(0.010)	0.039(0.374)
Diastolic BP	0.029 (0.501)	−0.017(0.697)	0.089(0.038)	0.036(0.407)
Serum TG	0.179 (0.000)	0.139(0.001)	0.122 (0.004)	0.039(0.369)
Serum TC	0.100 (0.020)	0.068(0.119)	0.193 (0.000)	0.091(0.036)
Serum HDL-c	−0.013 (0.759)	−0.007(0.878)	−0.038(0.375)	0.028(0.523)
Serum LDL-c	0.038 (0.386)	0.015(0.736)	0.176 (0.000)	0.059(0.171)
Atherogenic index (AI)	0.057 (0.190)	0.022(0.614)	0.139 (0.001)	−0.029(0.498)
Serum glucose	0.046 (0.291)	−0.022(0.614)	0.221 (0.000)	0.094(0.030)
Serum insulin	0.159 (0.000)	0.111(0.011)	0.155 (0.000)	0.137(0.002)
HOMA-IR	0.157 (0.000)	0.101(0.020)	0.185 (0.000)	0.143(0.001)
HOMA-β	0.147(0.001)	0.081(0.065)	0.216(0.000)	0.134(0.002)
QUICKI	−0.161 (0.000)	−0.104(0.017)	−0.185 (0.000)	−0.147(0.001)
**TMAO (μmol/L)**				
Weight	−0.050(0.247)	−0.056(0.202)	0.052(0.229)	0.032(0.456)
BMI	−0.033(0.453)	−0.065(0.136)	0.033(0.447)	−0.012(0.779)
Waist circumference	0.003(0.951)	−0.052(0.230)	0.067(0.121)	−0.009(0.827)
Hip circumference	0.011(0.798)	−0.031(0.471)	0.054(0.213)	−0.003(0.945)
Waist/hip ratio	−0.005(0.916)	−0.053(0.224)	0.045(0.298)	−0.020(0.652)
Systolic BP	0.091 (0.034)	0.055(0.206)	−0.004 (0.926)	−0.060(0.167)
Diastolic BP	0.067 (0.123)	0.016(0.719)	0.010 (0.817)	−0.014(0.745)
Serum TG	0.009 (0.828)	−0.012(0.778)	0.093 (0.031)	0.037(0.397)
Serum TC	0.036 (0.404)	0.018(0.680)	0.146 (0.001)	0.070(0.105)
Serum HDL-c	0.100 (0.020)	0.076(0.082)	0.021 (0.632)	0.052(0.228)
Serum LDL-c	0.019 (0.669)	0.012(0.777)	0.144 (0.001)	0.070(0.107)
Atherogenic index (AI)	−0.059 (0.176)	−0.055(0.207)	0.068 (0.115)	−0.002(0.955)
Serum glucose	0.101 (0.019)	0.000(0.994)	0.099 (0.021)	0.018(0.683)
Serum insulin	0.052 (0.232)	0.039(0.373)	0.056 (0.189)	0.063(0.147)
HOMA-IR	0.067 (0.121)	0.037(0.397)	0.071 (0.098)	0.061(0.162)
HOMA-β	0.078(0.073)	0.022(0.621)	0.090(0.036)	0.049(0.254)
QUICKI	−0.053 (0.220)	−0.022(0.611)	−0.067 (0.117)	−0.060(0.165)

*r1: correlation coefficient; r2: controlled for age, total calorie intake, physical activity level, medicine status, alcohol intake, smoking status; r3 controlled for age, total calorie intake, physical activity level, medicine status, alcohol intake, smoking status and menopausal staus; Statistical significance was set to p < 0.05*.

To further identify the associations, we performed linear regression analysis and the findings are presented in Table 3. After all the confounding factors were controlled, serum L-carnitine level was found to be positively correlated with serum TG level, serum insulin level, HOMA-IR index, and negatively correlated with QUICKI index in males (*p* < 0.05 for all). In females, serum L-carnitine level was positively correlated with weight, BMI, WC, hip circumference, serum TC, glucose level, insulin levels, HOMA-IR index, HOMA-β index, and negatively correlated with QUICKI index (*p* < 0.05 for all). Serum TMAO level was not associated with any component of MS in either females or males.

**Table 3:** Linear Regression analysis of serum L-carnitine, TMAO levels with metabolic syndrome related indexes^a^.

	**Men (*****n*** **=** **536)**			**Women (*****n*** **=** **545)**		
	***R*^**2**^**	β^*^	***P***	***R*^**2**^**	β^*^	***P***
**L-CARNITINE (μmol/L)**						
Weight	0.035	0.026	0.554	0.057	0.216	0.000
BMI	0.075	0.043	0.318	0.089	0.203	0.000
Waist circumference	0.138	0.030	0.459	0.132	0.155	0.001
Hip circumference	0.123	0.040	0.327	0.112	0.195	0.000
Waist/hip ratio	0.055	0.003	0.937	0.061	0.016	0.730
Systolic BP	0.041	0.033	0.446	0.080	0.040	0.388
Diastolic BP	0.107	−0.019	0.645	0.042	0.039	0.405
Serum TG	0.164	0.130	0.001	0.108	0.040	0.406
Serum TC	0.115	0.068	0.104	0.117	0.095	0.035
Serum HDL-c	0.085	−0.005	0.898	0.091	0.029	0.533
Serum LDL-c	0.090	0.017	0.692	0.106	0.064	0.161
Atherogenic index (AI)	0.154	0.021	0.603	0.095	0.032	0.481
Serum glucose	0.189	−0.016	0.684	0.150	0.097	0.028
Serum insulin	0.290	0.096	0.010	0.180	0.140	0.001
HOMA-IR	0.307	0.087	0.018	0.190	0.146	0.001
HOMA-β	0.302	0.071	0.056	0.190	0.137	0.002
QUICKI	0.305	−0.090	0.015	0.192	−0.150	0.001
**TMAO (μmol/L)**						
Weight	0.025	−0.059	0.188	0.020	0.035	0.438
BMI	0.066	−0.068	0.122	0.055	−0.010	0.822
Waist circumference	0.140	−0.052	0.216	0.113	−0.009	0.843
Hip circumference	0.123	−0.032	0.449	0.081	0.000	0.992
Waist/hip ratio	0.058	−0.054	0.216	0.061	−0.022	0.614
Systolic BP	0.043	0.053	0.236	0.080	−0.059	0.183
Diastolic BP	0.107	0.013	0.753	0.041	−0.014	0.763
Serum TG	0.147	−0.013	0.760	0.106	0.038	0.383
Serum TC	0.111	0.019	0.658	0.113	0.069	0.112
Serum HDL-c	0.090	0.077	0.077	0.092	0.050	0.252
Serum LDL-c	0.090	0.014	0.754	0.107	0.067	0.121
Atherogenic index (AI)	0.156	−0.053	0.206	0.093	−0.001	0.973
Serum glucose	0.188	0.002	0.969	0.141	0.022	0.613
Serum insulin	0.282	0.034	0.883	0.168	0.065	0.119
HOMA-IR	0.300	0.032	0.398	0.176	0.063	0.129
HOMA-β	0.298	0.019	0.613	0.177	0.052	0.209
QUICKI	0.298	−0.019	0.610	0.177	−0.062	0.137

a Models were multiple linear regression with statistical significant associations at p < 0.05. All analyses were controlled for age, total calorie intake, physical activity level, medicine status, alcohol intake, smoking status, and menopausal status. β, Unstandardized Beta;

β*, *standardized coefficient; R2, Adjusted R square*.

### Correlation Between Serum TMAO, L-Carnitine Levels, and MS Related Indexes in Subjects With Normal Serum Glucose Level

The correlations between serum L-carnitine and TMAO levels with MS-related indexes in subjects with normal glucose levels are shown in Table 4. In males, serum L-carnitine level was positively correlated with serum TG level (*r* = 0.126, *p* < 0.05), serum insulin level (*r* = 0.121, *p* < 0.05), HOMA-IR (*r* = 0.114, *p* < 0.05), and negatively correlated with QUICKI index (*r* = −0.113, *p* < 0.05). In females, serum L-carnitine level was positively correlated with weight (*r* = 0.164, *P* < 0.000), BMI (*r* = 0.157, *P* < 0.01), WC (*r* = 0.123, *P* < 0.01), hip circumference (*r* = 0.147, *P* < 0.01), serum TC (*r* = 0.093, *p* < 0.05), glucose levels (*r* = 0.092, *p* < 0.05), insulin levels (*r* = 0.139, *p* < 0.01), HOMA-IR index (*r* = 0.145, *p* < 0.01), HOMA-β index (*r* = 0.131, *p* < 0.01), and negatively correlated with QUICKI index (*r* = −0.146, *p* < 0.01). No significant relationships were found between serum TMAO level and MS related indexes.

**Table 4:** Partial correlations between serum L-Carnitine, TMAO levels with components of MS in females and males with normal serum glucose level.

	**Male (*****n*** **=** **400)**		**Female (*****n*** **=** **465)**	
	**r1(P)**	**r2(P)**	**r1(P)**	**r3(P)**
**L-CARNITINE (μmol/L)**				
Weight	0.030 (0.549)	0.017 (0.736)	0.174 (0.000)	0.164 (0.000)
BMI	0.053 (0.287)	0.033 (0.509)	0.210 (0.000)	0.157 (0.001)
Waist circumference	0.057 (0.254)	0.032 (0.522)	0.223 (0.000)	0.123 (0.009)
Hip circumference	0.050 (0.316)	0.025 (0.619)	0.224 (0.000)	0.147 (0.002)
Waist/hip ratio	0.046 (0.360)	0.033 (0.518)	0.097 (0.037)	0.017 (0.712)
Systolic BP	0.067 (0.183)	0.055 (0.276)	0.072 (0.121)	0.007 (0.882)
Diastolic BP	0.027 (0.588)	0.003 (0.956)	0.052 (0.267)	0.002 (0.961)
Serum TG	0.149 (0.003)	0.126 (0.013)	0.106 (0.023)	0.029 (0.543)
Serum TC	0.104 (0.037)	0.072 (0.154)	0.201 (0.000)	0.093 (0.047)
Serum HDL-c	−0.034 (0.498)	−0.038 (0.458)	−0.051 (0.276)	−0.006 (0.904)
Serum LDL-c	0.068 (0.175)	0.040 (0.435)	0.191 (0.000)	0.078 (0.98)
Atherogenic index (AI)	0.076 (0.130)	0.051 (0.310)	0.149 (0.001)	0.053 (0.257)
Serum glucose	−0.016 (0.746)	−0.040 (0.427)	0.219 (0.000)	0.092 (0.049)
Serum insulin	0.138 (0.006)	0.121 (0.016)	0.123 (0.008)	0.139 (0.003)
HOMA-IR	0.134 (0.007)	0.114 (0.024)	0.149 (0.001)	0.145 (0.002)
HOMA-β	0.119 (0.017)	0.093 (0.066)	0.182 (0.000)	0.131 (0.005)
QUICKI	−0.134 (0.007)	−0.113 (0.026)	−0.147 (0.002)	−0.146 (0.002)
**TMAO (μmol/L)**				
Weight	−0.073 (0.147)	−0.072 (0.157)	0.049 (0.289)	0.039 (0.410)
BMI	−0.066 (0.190)	−0.084 (0.096)	0.053 (0.254)	0.011 (0.813)
Waist circumference	−0.022 (0.658)	−0.062 (0.223)	0.077 (0.097)	−0.002 (0.964)
Hip circumference	−0.027 (0.587)	−0.053 (0.296)	0.059 (0.203)	0.004 (0.939)
Waist/hip ratio	0.007 (0.890)	−0.036 (0.474)	0.054 (0.250)	−0.014 (0.773)
Systolic BP	0.072 (0.150)	0.063 (0.211)	0.007 (0.874)	−0.051 (0.278)
Diastolic BP	0.044 (0.380)	0.015 (0.760)	0.024 (0.603)	−0.003 (0.948)
Serum TG	0.020 (0.696)	0.031 (0.540)	0.129 (0.005)	0.072 (0.127)
Serum TC	0.076 (0.130)	0.063 (0.216)	0.177 (0.000)	0.085 (0.070)
Serum HDL-c	0.106 (0.034)	0.075 (0.140)	0.012 (0.793)	0.041 (0.380)
Serum LDL-c	0.054 (0.285)	0.042 (0.411)	0.169 (0.000)	0.083 (0.077)
Atherogenic index (AI)	−0.035 (0.480)	−0.025 (0.628)	0.099 (0.033)	0.023 (0.632)
Serum Glucose	−0.012 (0.812)	−0.052 (0.301)	0.124 (0.007)	0.029 (0.536)
Serum Insulin	−0.026 (0.604)	0.000 (0.994)	0.055 (0.240)	0.072 (0.125)
HOMA-IR	−0.027 (0.593)	−0.006 (0.904)	0.070 (0.131)	0.072 (0.123)
HOMA-β	−0.030 (0.549)	−0.024 (0.633)	0.095 (0.041)	0.061 (0.194)
QUICKI	0.031 (0.534)	0.012 (0.819)	−0.064 (0.166)	−0.068 (0.144)

*r1: correlation coefficient; r2: controlled for age, total caloric intake, physical activity level, medicine status, alcohol intake, smoking status; r3 controlled for age, total calorie intake, physical activity level, medicine status, alcohol intake, smoking status and menopausal status; Statistical significance was set to p < 0.05*.

Linear regression analysis was performed to further identify the correlations between serum L-carnitine, TMAO levels, and MS in subjects with normal serum glucose levels, and the findings are presented in Table 5. After all confounding factors were controlled, serum L-carnitine level was positively correlated with serum TG level, serum insulin level, HOMA-IR index and negatively correlated with QUICKI index in males (*p* < 0.05 for all). In females, serum L-carnitine level was positively correlated with weight, BMI, WC, hip circumference, serum TC, glucose levels, insulin levels, HOMA-IR index, HOMA-β index and negatively correlated with QUICKI index (*p* < 0.05 for all). Serum TMAO level was not associated with any component of the MS in both females and males.

**Table 5:** Linear Regression analysis of serum L-carnitine, TMAO levels with MS related indexes in subjects with normal fasting glucose levels^a^.

	**Male (*****n*** **=** **400)**			**Female (*****n*** **=** **465)**		
	***R*^**2**^**	β^*^	***P***	***R*^**2**^**	β^*^	***P***
**L-CARNITINE (μmol/L)**						
Weight	0.030	0.018	0.716	0.030	0.181	0.000
BMI	0.073	0.034	0.489	0.071	0.170	0.001
Waist circumference	0.143	0.031	0.505	0.129	0.128	0.009
Hip circumference	0.124	0.026	0.576	0.102	0.156	0.002
Waist/hip ratio	0.065	0.030	0.546	0.067	0.020	0.693
Systolic BP	0.018	0.048	0.338	0.049	0.007	0.893
Diastolic BP	0.106	−0.001	0.978	0.028	0.003	0.960
Serum TG	0.181	0.114	0.014	0.112	0.030	0.543
Serum TC	0.152	0.071	0.128	0.130	0.096	0.047
Serum HDL-c	0.083	−0.033	0.495	0.090	−0.006	0.903
Serum LDL-c	0.121	0.041	0.386	0.112	0.082	0.094
Atherogenic index (AI)	0.182	0.048	0.296	0.106	0.055	0.258
Serum glucose	0.057	−0.038	0.445	0.129	0.096	0.047
Serum insulin	0.278	0.104	0.016	0.150	0.143	0.003
HOMA-IR	0.277	0.098	0.023	0.151	0.149	0.002
HOMA-β	0.245	0.082	0.064	0.137	0.135	0.005
QUICKI	0.270	−0.097	0.025	0.151	−0.150	0.002
**TMAO (μmol/L)**						
Weight	0.035	−0.072	0.158	0.005	0.041	0.407
BMI	0.079	−0.082	0.097	0.048	0.011	0.817
Waist circumference	0.146	−0.058	0.225	0.116	−0.003	0.956
Hip circumference	0.126	−0.050	0.301	0.082	0.004	0.937
Waist/hip ratio	0.065	−0.036	0.469	0.068	−0.015	0.758
Systolic BP	0.019	0.060	0.241	0.050	−0.051	0.294
Diastolic BP	0.106	0.012	0.799	0.029	−0.002	0.970
Serum TG	0.169	0.028	0.554	0.113	0.070	0.135
Serum TC	0.151	0.062	0.195	0.127	0.085	0.069
Serum HDL-c	0.088	0.076	0.127	0.091	0.043	0.361
Serum LDL-c	0.121	0.042	0.383	0.112	0.081	0.084
Atherogenic index (AI)	0.180	−0.022	0.633	0.102	0.020	0.679
Serum glucose	0.058	−0.051	0.311	0.122	0.028	0.553
Serum insulin	0.267	0.000	0.994	0.139	0.071	0.125
HOMA-IR	0.268	−0.005	0.907	0.137	0.071	0.124
HOMA-β	0.239	−0.021	0.639	0.124	0.060	0.198
QUICKI	0.261	0.010	0.821	0.136	−0.068	0.146

a Models were multiple linear regression with statistical significant associations at p < 0.05. All analyses were controlled for age, total calorie intake, physical activity, BF%, medicine status, alcohol intake, smoking status and menopausal status. β, Unstandardized Beta;

β*, *standardized coefficient; R2, Adjusted R square*.

### Correlation Between Serum TMAO, L-Carnitine Levels, and MS Related Indexes in Subjects With Hyperglycemia

The correlations between serum L-carnitine and TMAO levels with MS related indexes in subjects with hyperglycemia are presented in Table 6. In males, serum L-carnitine level was positively correlated with serum TG level (*r* = 0.180, *p* < 0.05). No significant relationships were found between serum L-carnitine level and MS related indexes in females.

**Table 6:** Partial correlations between serum L-carnitine, TMAO levels with components of MS in females and males with hyperglycemia.

	**Male (*****n*** **=** **136)**		**Female (*****n*** **=** **79)**	
	**r1(P)**	**r2(P)**	**r1(P)**	**r3(P)**
**L-CARNITINE (μmol/L)**				
Weight	0.058(0.510)	0.051(0.564)	0.193 (0.093)	0.213(0.074)
BMI	0.108(0.217)	0.079(0.378)	0.157 (0.174)	0.155(0.197)
Waist circumference	0.081(0.352)	0.019(0.833)	0.130(0.260)	0.143(0.234)
Hip circumference	0.160(0.066)	0.093(0.295)	0.210(0.066)	0.197(0.099)
Waist/hip ratio	−0.113(0.196)	−0.126(0.157)	−0.41(0.724)	0.005(0.966)
Systolic BP	0.018(0.831)	−0.021(0.816)	0.103(0.366)	0.108(0.378)
Diastolic BP	−0.005(0.956)	−0.116(0.194)	0.099(0.384)	0.174(0.153)
Serum TG	0.218(0.011)	0.180(0.044)	0.040 (0.726)	−0.005(0.965)
Serum TC	0.067(0.440)	0.105(0.244)	0.030(0.794)	0.094(0.444)
Serum HDL-c	0.052(0.551)	0.124(0.166)	0.130(0.252)	0.266(0.027)
Serum LDL-c	−0.045(0.601)	−0.004(0.965)	−0.041(0.718)	−0.010(0.934)
Atherogenic index (AI)	−0.016(0.849)	−0.052(0.561)	−0.101(0.377)	−0.161(0.186)
Serum glucose	−0.100(0.245)	−0.124(0.167)	−0.040(0.724)	−0.105(0.391)
Serum insulin	0.138(0.109)	0.046(0.610)	0.084 (0.464)	−0.066(0.589)
HOMA-IR	0.115(0.182)	0.020(0.820)	0.070(0.538)	−0.084(0.492)
HOMA-β	0.085(0.324)	−0.010(0.912)	0.056 (0.626)	−0.098(0.425)
QUICKI	−0.133(0.123)	−0.039(0.667)	−0.099(0.385)	0.055(0.651)
**TMAO (μmol/L)**				
Weight	0.014(0.871)	0.003(0.970)	0.022(0.851)	0.005(0.969)
BMI	0.044(0.612)	0.001(0.995)	−0.116(0.315)	−0.122(0.311)
Waist circumference	0.035(0.693)	−0.007(0.940)	−0.034(0.771)	−0.056(0.644)
Hip circumference	0.109(0.213)	0.044(0.619)	−0.025(0.831)	−0.029(0.813)
Waist/hip ratio	−0.127(0.144)	−0.097(0.297)	−0.051(0.659)	−0.089(0.461)
Systolic BP	0.086(0.321)	0.022(0.810)	−0.123(0.281)	−0.142(0.246)
Diastolic BP	0.096(0.265)	−0.022(0.805)	−0.105(0.357)	−0.088(0.472)
Serum TG	−0.095(0.271)	−0.139(0.122)	−0.134(0.240)	−0.145(0.235)
Serum TC	−0.110(0.201)	−0.069(0.443)	−0.029(0.798)	0.008(0.949)
Serum HDL-c	0.079(0.360)	0.096(0.287)	0.085(0.454)	0.137(0.262)
Serum LDL-c	−0.080(0.352)	−0.027(0.765)	−0.015(0.896)	−0.007(0.956)
Atherogenic index (AI)	−0.149(0.083)	−0.129(0.150)	−0.150(0.188)	−0.154(0.205)
Serum glucose	0.153(0.075)	0.088(0.328)	0.074(0.519)	0.075(0.540)
Serum insulin	0.211(0.014)	0.172(0.045)	0.028(0.809)	0.074(0.544)
HOMA-IR	0.235(0.006)	0.188(0.035)	0.041(0.720)	0.085(0.486)
HOMA-β	0.252(0.003)	0.197(0.027)	0.053(0.642)	0.092(0.452)
QUICKI	−0.221(0.010)	−0.175(0.049)	−0.044(0.699)	−0.092(0.450)

*r1: correlation coefficient; r2: controlled for age, total calorie intake, physical activity level, medicine status, alcohol intake, smoking status; r3 controlled for age, total calorie intake, physical activity level, medicine status, alcohol intake, smoking status and menopausal status; Statistical significance was set to p < 0.05*.

Serum TMAO level was positively related with insulin levels (*r* = 0.172, *p* < 0.05), HOMA-IR index (*r* = 0.188, *p* < 0.05), HOMA-β index (*r* = 0.197, *p* < 0.05) and negatively correlated with QUICKI index (*r* = −0.175, *p* < 0.05. No significant relationships were found between serum TMAO level and MS related indexes in females.

Linear regression analysis was also performed to further identify the correlations between serum L-carnitine, TMAO levels, and MS in subjects with hyperglycemia, and the findings are presented in Table 7. In males, after all the confounding factors were controlled, serum L-carnitine level was positively correlated with serum TG level (*p* < 0.05). While serum TMAO level was positively related to serum insulin levels, HOMA-IR index and HOMA-β index, it was negatively correlated with QUICKI index (*p* < 0.05 for all). In females, neither serum L-carnitine nor TMAO levels were associated with any component of MS.

**Table 7:** Linear regression analysis of serum L-carnitine, TMAO levels with metabolic syndrome related indexes in subjects with hyperglycemia^a^.

	**Men (*****n*** **=** **536)**			**Women (*****n*** **=** **545)**		
	***R*^**2**^**	β^*^	***P***	***R*^**2**^**	β^*^	***P***
**L-CARNITINE (μmol/L)**						
Weight	0.010	0.066	0.477	0.112	0.233	0.065
BMI	0.009	0.098	0.291	0.072	0.169	0.190
Waist circumference	0.038	0.037	0.681	−0.023	0.163	0.227
Hip circumference	0.061	0.114	0.205	0.034	−0.028	0.813
Waist/hip ratio	0.004	−0.127	0.172	−0.030	−0.090	0.461
Rsystolic BP	-0.010	−0.034	0.716	0.007	0.112	0.410
Diastolic BP	0.123	−0.118	0.172	−0.047	0.203	0.147
Serum TG	0.041	0.183	0.044	0.008	−0.013	0.924
Serum TC	0.057	0.102	0.252	0.113	0.097	0.450
Serum HDL-c	0.089	0.003	0.180	0.061	0.288	0.031
Serum LDL-c	0.061	−0.003	0.975	0.046	−0.005	0.970
Atherogenic index (AI)	0.066	−0.054	0.548	−0.070	−0.168	0.234
Serum glucose	0.126	−0.117	0.175	−0.035	−0.114	0.411
Serum insulin	0.184	0.040	0.632	0.120	−0.050	0.696
HOMA-IR	0.209	0.017	0.203	0.096	−0.070	0.587
HOMA-β	0.223	−0.011	0.895	0.069	−0.087	0.508
QUICKI	0.218	−0.035	0.668	0.114	0.041	0.748
**TMAO (μmol/L)**						
Weight	0.006	0.005	0.960	0.066	0.010	0.931
BMI	0.000	0.002	0.981	0.062	−0.116	0.324
Waist circumference	0.037	−0.005	0.955	−0.043	−0.054	0.661
Hip circumference	0.051	0.048	0.02	0.021	−0.026	0.827
Waist/hip ratio	-0.001	−0.102	0.282	−0.044	−0.088	0.457
Systolic BP	-0.011	0.021	0.826	0.020	−0.149	0.218
Diastolic BP	0.110	−0.024	0.785	−0.070	−0.105	0.405
Serum TG	0.029	−0.148	0.114	0.027	−0.134	0.265
Serum TC	0.052	−0.074	0.418	0.105	−0.008	0.946
Serum HDL-c	0.084	0.093	0.302	0.007	0.114	0.349
Serum LDL-c	0.061	−0.029	0.753	0.046	−0.018	0.882
Atherogenic index (AI)	0.079	−0.132	0.145	−0.071	−0.145	0.248
Serum glucose	0.121	0.092	0.299	−0.039	0.080	0.518
Serum insulin	0.206	0.161	0.047	0.127	0.095	0.402
HOMA-IR	0.237	0.175	0.036	0.104	0.106	0.358
HOMA-β	0.253	0.183	0.027	0.076	0.113	0.335
QUICKI	0.240	−0.162	0.041	0.125	−0.111	0.331

a Models were multiple linear regression with statistical significant associations at p < 0.05. All analyses were controlled for age, total calorie intake, physical activity level, BF%, medicine status, alcohol intake, smoking status and menopausal status. β, Unstandardized Beta;

β*, *standardized coefficient; R2, Adjusted R square*.

## Discussion

In this relatively large population-based cross-sectional study, we investigated the associations of serum L-carnitine and TMAO levels with MS related indexes, including obesity, blood pressure, serum lipids, serum glucose, and IR-related indexes after controlling for major confounding factors. Significant positive associations were identified in a number of physiological and pathological conditions. Firstly, we discovered that there was a positive correlation between serum L-carnitine and TMAO levels with MS profiles based on serum glucose status. Moreover, we revealed that some associations are gender specific. Briefly, serum L-carnitine level was positively correlated with serum TG level, serum insulin level, IR in males with normal fasting glucose levels and positively correlated with only serum TG level in those with hyperglycemia. In females, positive correlations were found between serum L-carnitine level and obesity, serum TC level, serum glucose level, serum insulin levels, IR in females with normal fasting glucose levels, while none was found in those with hyperglycemia. Serum TMAO level was positively correlated with serum insulin level and IR in males with hyperglycemia only.

The present study has several strengths. Firstly, a broad involvement of MS related components. MS is comprised of multiple risk factors for T2D and CVD, including obesity, hypertension, dyslipidemia, hyperglycemia and IR with obesity and IR as the source of pathogenesis (28). In the present study, all five categories of MS related components (obesity, blood pressure, serum lipid levels, fasting glucose, and IR) were systematically studied. Due to the relationship between MS and CVD, atherogenic index was calculated and included in the analysis as well (29). In addition, the systematic control of major confounding factors makes the findings more reliable. In a large population-based study, identifying the primary confounding factors and properly controlling their effects are essential steps for obtaining reliable results. MS is a complicated pathological condition with various factors involved (30), especially age and gender (7, 30). Calorie intake and physical activity level, major factors in keeping the balance of energy obtained and consumed, can strongly influence hypertension (31), glucose and lipid metabolism (7), and insulin sensitivity (32). Moreover, medicine status, alcohol intake, smoking status, and menopausal status were taken into consideration in analyses. Medication use, alcohol consumption, and smoking status are recognized as potential covariates that may influence energy intake, hypertension risk, and insulin sensitivity (33, 34). Menopause is related to changes in sex hormones that may affect MS profiles in females (35). In this study, all these factors that can potentially affect the relationships between serum L-carnitine and TMAO levels with MS profiles were identified and controlled.

The most notable finding in the present study is the positive correlation of serum L-carnitine levels and TMAO levels with MS profiles. Moreover, the significant correlations are gender specific and depend on serum glucose status. L-carnitine is an important metabolite involved in fatty acid metabolism and has been used as an ergogenic aid in treatment of weight loss (36). While, herein, we found unfavorable associations between serum L-carnitine levels with MS profiles. In subjects with normal fasting glucose levels, serum L-carnitine was found to be positively correlated with serum TG level, serum insulin level, IR in males and with obesity measurements, serum TC level, serum glucose levels, serum insulin levels, and IR in females, while in subjects with hyperglycemia there was only positive correlation between serum L-carnitine and TG levels in males. Association studies regarding serum L-carnitine levels and MS are rare. To date, only one cross-sectional study examined the correlations between serum L-carnitine levels and MS profile in 118 healthy women with singleton term pregnancy (16). The study identified significant negative correlations between serum L-carnitine with pre-pregnancy body weight, BMI and serum TG levels (16). Due to the unusual metabolic and energy status during pregnancy, the difference is hard to explain and may not be contradictory. However, numerous interventional studies have been performed and have shown weight loss and anti-hyperlipidemia effect of dietary L-carnitine supplementation in both humans and animals (36). Dietary treatment with L-carnitine also alleviates IR in high fructose-fed rodents (15). These studies show results apparently inconsistent with ours. While L-carnitine is not only acquired through dietary intake, it is also synthesized *de novo* from L-lysine and L-methionine (36). For this reason, L-carnitine is not recognized as an essential dietary nutrient and no recommended nutrient intakes (RNI) are set (37). Moreover, L-carnitine supplementation and L-carnitine acquired through diet also represent two significantly different situations. Getting more L-carnitine through diet alone means also taking in more meat protein and fat, that carry a negative impact on health, while, L-carnitine supplementation avoids these downsides. Hence, the correlations between dietary L-carnitine supplementation and serum L-carnitine levels with MS may be different and requires further investigation. In addition, L-carnitine is reported to decrease hypertension (38). However, no significant correlations were found between serum L-carnitine level and blood pressure in our study.

Serum TMAO level was only positively correlated with serum insulin level and IR in hyperglycemic males in our study. TMAO is a gut microbiota generated metabolite which can be generated from dietary L-carnitine or other TMA containing components in food. Recent studies reported that elevated TMAO levels were observed in various metabolic diseases, including patients with CVD (39), T2DM (40) or chronic kidney diseases (41) and rodents with obesity (42). Cross-sectional studies regarding the relationship between serum TMAO level and MS profiles are rare. Chen et al. reported serum TMAO level positively associated with serum TG, TC, LDL-c levels and development of NAFLD in 1,628 community-based adults (43). A recent cross-sectional study by Latkovskis et al. indicated that serum TMAO level was significantly related with IR in 300 patients with increased CVD risk (13). All these reports indicate that TMAO plays a potential role in the progression of various chronic diseases. Our previous study in male mice proved that supplementary dietary TMAO exacerbated IR in high fat diet induced obese mice by inducing chronic inflammation in adipose tissue and blocking insulin signaling pathway conduction, while it has no effect on normal mice (12). These findings are consistent with the observations of our current study. No correlations were identified between serum TMAO levels and MS profiles in subjects with normal serum glucose level, while significant associations were identified between serum TMAO levels and IR in hyperglycemia males. Our findings suggested that potentially harmful effects of TMAO may be more pronounced in metabolically abnormal subjects, and may not be evident or easily detected in milder cases of MS or potentially earlier on in the condition onset. Moreover, although studies in rodents reported that serum TMAO levels were elevated in obese subjects (43), we did not find any notable correlations between serum TMAO level and obesity measures. This suggests that other complex factors impacting on serum TMAO level may be at play, which would require further studies.

Some limitations should be acknowledged here. First, this study is of cross-sectional design and we could not build causal relationships between serum L-carnitine or TMAO levels and components of MS. Further longitudinal or interventional studies are warranted. Secondly, the number of subjects in hyperglycemia groups are limited due to the composition of our cohort and difficulty in obtaining essential data. A larger study is needed to confirm the significant relationships between serum L-carnitine or TMAO levels and major outcomes. Lastly, although multiple factors were systematically controlled in our analysis, genetics or unknown residual factors could not be completely ruled out but might be of significance in reconciling our findings with those of others.

## Conclusion

In conclusion, in general Newfoundland population, we provide solid evidence for the first time that serum L-carnitine level is significantly associated with an unfavorable MS profile, mainly serum TG levels, serum insulin level, IR in male subjects with normal serum glucose level and obesity, serum TC level, serum glucose level, serum insulin levels, and IR in females with normal serum glucose level. Serum TMAO level was positively associated with IR related indexes in hyperglycemic males. The totality of these unfavorable relationships were independent of age, total calorie intake, physical activity level, medication use, smoking status, alcohol consumption, and menopausal status. Our findings reveal novel insights into the relationships between serum TMAO and its related metabolic precursors in human health.

## Data Availability

All datasets generated for this study are included in the manuscript and/or the supplementary files.

## Author Contributions

GS and XG conceived and designed the experiments. XG and ER performed the experiments. XG and YT analyzed the data. XG draft the paper which was reviewed and modified by ER, HZ, and GS. All authors have read and approved the final manuscript.

### Conflict of Interest Statement

The authors declare that the research was conducted in the absence of any commercial or financial relationships that could be construed as a potential conflict of interest.
